# Closing the loop: autonomous experiments enabled by machine-learning-based online data analysis in synchrotron beamline environments

**DOI:** 10.1107/S160057752300749X

**Published:** 2023-10-17

**Authors:** Linus Pithan, Vladimir Starostin, David Mareček, Lukas Petersdorf, Constantin Völter, Valentin Munteanu, Maciej Jankowski, Oleg Konovalov, Alexander Gerlach, Alexander Hinderhofer, Bridget Murphy, Stefan Kowarik, Frank Schreiber

**Affiliations:** a Institut für Angewandte Physik, Universität Tübingen, Auf der Morgenstelle 10, 72076 Tübingen, Germany; b Physikalische und Theoretische Chemie, Universität Graz, Heinrichstrasse 28, 8010 Graz, Austria; c Institut für Experimentelle und Angewandte Physik, Universität Kiel, Leibnizstrasse 19, 24118 Kiel, Germany; d ESRF – The European Synchrotron, 71 Avenue des Martyrs, CS 40220, 38043 Grenoble Cedex 9, France; NSRRC, Taiwan

**Keywords:** machine learning, reflectometry, autonomous experiments, beamline control, XRR, closed-loop control

## Abstract

Recently, there has been significant interest in applying machine-learning (ML) techniques to the automated analysis of X-ray scattering experiments, due to the increasing speed and size at which datasets are generated. ML-based analysis presents an important opportunity to establish a closed-loop feedback system, enabling monitoring and real-time decision-making based on online data analysis. In this study, the incorporation of a combined one-dimensional convolutional neural network (CNN) and multilayer perceptron that is trained to extract physical thin-film parameters (thickness, density, roughness) and capable of taking into account prior knowledge is described. ML-based online analysis results are processed in a closed-loop workflow for X-ray reflectometry (XRR), using the growth of organic thin films as an example. Our focus lies on the beamline integration of ML-based online data analysis and closed-loop feedback. Our data demonstrate the accuracy and robustness of ML methods for analyzing XRR curves and Bragg reflections and its autonomous control over a vacuum deposition setup.

## Abstract 2

A machine-learning-based closed-loop solution for reflectometry analysis in synchrotron beamline operation utilizing online data analysis is presented. This work focuses on the perspective of visiting facility users and strategies to provide an elementary data analysis in real time during the experiment without introducing the additional software dependencies in the beamline control software environment.

## 1. Introduction

X-ray user facilities are amongst the largest scientific data producers in the world (Yanxon *et al.*, 2023 ▸). While experiments performed at these facilities cover an extensive range of multi-disciplinary sciences, they typically share a common data generation pattern, namely precisely positioning a specimen in the path of the X-ray beam and recording data (*e.g.* radiation scattered by the sample) captured by dedicated detectors (*e.g.* diffraction, imaging, spectroscopy) further downstream. Recent advances in accelerator development such as fourth-generation synchrotron light sources (Raimondi, 2016 ▸) and innovative detector technology (*e.g.* higher acquisition rates and larger area detector dimensions) lead to continuously increasing data volume of these datasets that are more and more difficult to handle. In fact, in order to manage acquisition, analysis and storage of these, specific solutions developed at the facilities (Guijarro *et al.*, 2018 ▸; Allan *et al.*, 2019 ▸; Novelli *et al.*, 2023 ▸) or in data-driven national and international collaborations, *e.g.* DAPHNE4NFDI (Barty *et al.*, 2023 ▸) or PaNOSC (Carboni, 2022 ▸; see also https://www.panosc.eu/), have been put in place, addressing these challenges.

Through the described technological advances and newly deployed infrastructure at modern beamlines and facilities, the way experiments are performed has changed significantly towards more and more data-intense and data-driven experiments, increasingly relying on machine-learning (ML) based approaches for data analysis (Chen *et al.*, 2021 ▸; Campbell *et al.*, 2021 ▸). While facility instruments used to be basically fully isolated systems, often posing complications for visiting facility users to integrate sample-environment-related equipment or data sources into the beamline data acquisition system, this has changed rapidly with the recent developments in many aspects. This process is generally encouraged as the value of freely available, complete, augmented and documented FAIR datasets (Wilkinson *et al.*, 2016 ▸; Scheffler *et al.*, 2022 ▸) is recognized. These datasets are specifically valuable for ML activities and enable novel, previously impossible experiments that fully utilize modern infrastructure.

This paper introduces an approach for integrating real-time X-ray scattering and diffraction data analysis into a closed-loop control system that actively adjusts sample environment parameters. This approach unlocks exciting possibilities for conducting experiments using synchrotron radiation that unveil new insights into the underlying physics. While several approaches focusing on automatic and optimized data acquisition based on, for example, Gaussian processes defining an acquisition function have been reported (Teixeira Parente *et al.*, 2023 ▸; McDannald *et al.*, 2022 ▸; Kusne *et al.*, 2020 ▸; Yager *et al.*, 2023 ▸; Noack *et al.*, 2021 ▸), in this study we describe a feedback mechanism relying on actual online data analysis, extracting physical parameters on-the-fly to control an *in situ* experiment.

We demonstrate the seamless integration of user-developed ML code with the beamline control infrastructure, enabling real-time data analysis and integrated archiving of the analyzed results improving the dataset quality with respect to FAIR principles. Additionally, focusing on reflectometry as a case study, we provide a concise overview of a ML-based approach for predicting thin-film parameters in both single-layer and multilayer structures. This use case convincingly demonstrates the main advantages of using ML in this context. The ML approach gives reliable fit results both for simple two- to three-layer models as well as for complex multilayer models in the millisecond regime. The combined speed and reliability of the ML approach could not be achieved by simple fitting scripts or with reliance on human supervision.

## 2. Methods and data

Several recent publications highlight the use of ML in synchrotron and neutron beamline environments (Noack *et al.*, 2021 ▸; Yager *et al.*, 2023 ▸; Beaucage & Martin, 2023 ▸; Szymanski *et al.*, 2023 ▸; Kandel *et al.*, 2023 ▸; Suzuki *et al.*, 2020 ▸). These developments have mainly been initiated at large-scale facilities or laboratories using privileged access rights to integrate additional software code into beamline software environments. However, often user- and sample-specific ML techniques are needed in experiments. Therefore, this study explores potential approaches to deploy ML-based real-time analysis code at large-scale facilities (X-ray and neutron sources) that are relevant also beyond the specific experiment or research facility by highlighting general deployment and integration strategies of user-developed ML codes at such facilities. Where possible, we follow guidelines based on community initiatives such as PaNOSC, ExPaNDS (https://www.panosc.eu/), DAPHNE4NFDI (Barty *et al.*, 2023 ▸) or MLExchange (Zhao *et al.*, 2022 ▸) (for details see the glossary in Appendix *A* [App appa]).

### 2.1. Software environments and structural requirements

**Figure 1:**
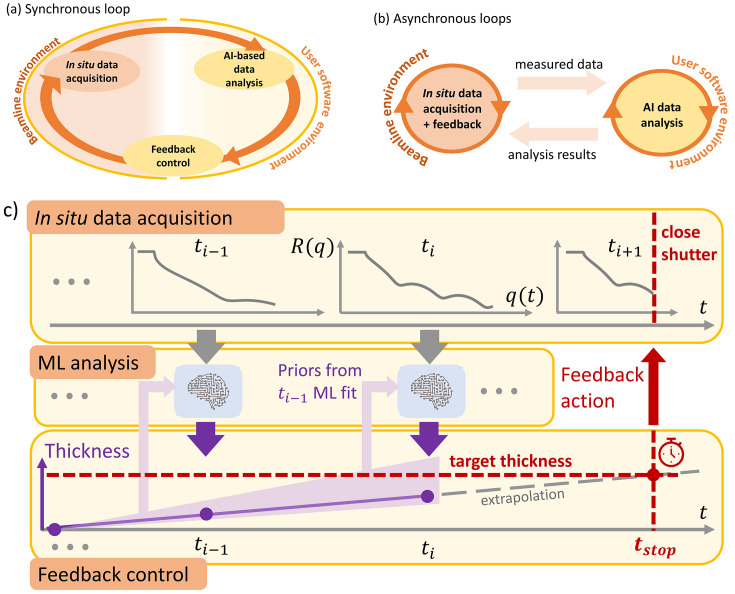
Illustration of a synchronous (*a*) and asynchronous (*b*) control loop with tasks split across a beamline, and user software environment. (*c*) Simplified schematic of the measurement protocol consisting of XRR acquisition, instantaneous ML-based online data analysis and thin-film growth control based on closed-loop feedback. Based on preceding measurements a time estimate to reach the predefined thickness is established to stop the growth process.

Before discussing the architecture in detail, it is worth describing the complete data acquisition and handling process on a conceptual level and experiment-specific level summarized in Fig. 1 ▸. First, in Figs. 1 ▸(*a*) and 1(*b*) we aim at separating conceptually feedback architectures operating in synchronous and asynchronous closed loops. In the first case, Fig. 1 ▸(*a*), data analysis is subsequently taking place after data acquisition (*e.g.* a scan) has finished. This simple mode of operation might be preferable in cases where data analysis is much faster than the actual data acquisition process. In the second case, Fig. 1 ▸(*b*), data analysis might also follow data acquisition; however, the data acquisition part does not wait for analysis results but keeps measuring, *i.e.* beamline motors keep moving while data are still analyzed and results are transferred into the appropriate feedback action on-the-fly.

Independent of the choice regarding synchronous or asynchronous control we can identify one part in these control loops that is strictly attached to beamline operation and as such also to a beamline software environment. A second part is potentially user-developed code that is to be executed in a user software environment. Software interfaces that enable the embedding of user-developed code into the beamline software environment are crucial for the presented experiment and – in our view – most likely also for many future experiments yet to come. To ensure operational stability of instruments with rapidly changing users there is an inherent dilemma from the facility point of view, since allowing modifications to the beamline’s control software environment by one user puts the experiments of subsequent users at risk. Therefore, as a matter of best practice, well isolated software environments for beamline control and user code are crucial and one must find suitable methods to exchange data between these environments and software processes [Figs. 1 ▸(*a*) and 1(*b*)]. From a practical point of view, such an approach also removes the burden of dealing with incompatibilities in software dependencies (*e.g.* versions of specific Python packages) that are used on both sides.

Approaching the specific experiment that is conducted in this study at high abstraction level, the acquisition of every X-ray reflectometry (XRR) profile is followed by immediate, ML-based online data analysis. In this step, prior knowledge, *e.g.* boundary conditions on the thin-film thickness that may change during the course of the growth process, is taken into account. The extracted thin-film parameters are used to estimate the time at which a predefined thickness is reached and the termination of the growth process is triggered – serving as the most simple example of a closed-loop feedback.

On a technical level and from an experimentalists’ point of view, there are two main types of data generators: (i) area detectors (specifically a Dectris Pilatus 300k and a MaxiPix 2×2 with a CdTe sensor) controlled via LIMA (Petitdemange *et al.*, 2018 ▸), and (ii) motor positioners. Experiment control is performed via *BLISS* (Guijarro *et al.*, 2018 ▸) which also handles saving of the collected data in the NeXus-HDF5 file format (Könnecke *et al.*, 2015 ▸) and triggers the ingestion of data and metadata into the facility data catalog.

### 2.2. Distributed online data analysis and closed-loop beamline integration

We identified different ‘hooks’ that typically exist in a modern beamline environment to achieve this inter-process communication between the beamline control software and user-supplied data analysis code, focusing on various levels of integration and portability between different light sources targeting universal concepts to make user-developed ML analysis available from the beamline control system. Furthermore, we aim at increasing the reusability of the produced datasets by enriching facility-produced datasets with user-provided real-time data analysis results (data + metadata), working towards datasets in facility data catalogs that are FAIR ‘immediately’ after acquisition without manually revisiting the dataset.

Modern beamline control systems, such as *BLISS* at the ESRF (Guijarro *et al.*, 2018 ▸) and *Bluesky* (Allan *et al.*, 2019 ▸), offer dedicated frameworks to access data in the beamline environment, *e.g.* through ‘publishing’ the data through an integrated in-memory database or via direct access to data- and event-streams produced by the acquisition process. Evidently, these are hooks that could be used to integrate online data analysis (see the example in Section SI-3 of the supporting information); however, these are usually tightly coupled to a specific facility and typically introduce a number of critical dependencies into the user-supplied code.

Another relatively simple option for external users to handle and test prior to the experiment is to integrate on the level of the SCADA (supervisory control and data acquisition) used at the respective facility. The two most spread systems in this field are TANGO controls (Götz *et al.*, 2022 ▸; see also https://www.tango-controls.org) and EPICS (https://epics-controls.org/). In the context of this study, we evaluated the integration via TANGO that is used at a sizable fraction of, mostly European, synchrotron facilities such as ESRF, DESY, SOLEIL, MAX IV and ALBA, but also in the broader scientific community such as the square kilometre array telescope. Data transfer from the beamline control system via the available SCADA system forms part of the most common operations performed on the control system level and thus does not induce any additional software dependencies for the beamline control software environment. To our knowledge this study represents a first attempt to combine ML/AI and TANGO.

Further, there is also the possibility to rely on workflow engines offered by facilities; for example, at the ESRF documented by De Nolf *et al.* (2022 ▸). However, care must be taken with respect to the online analysis capabilities of these systems since they inherently rely on queue systems (job schedulers for batch processing) and thus may introduce additional delays under heavy load.

When it comes specifically to ML/AI pipelines, there is also potential to rely on standardized solutions that specifically fit the needs of handling larger ML models (*e.g.* NVIDIA Triton Inference Server) and thus abstract even beyond the specificity of beamline environments.

Splitting beamline control and ML-based online data analysis in terms of infrastructure makes sense also when looking at the different hardware requirements of the two processes. While the beamline control process links to beamline instrumentation, it is physically tied to hardware that is available on the beamline itself. The ML data analysis process, which requires a powerful GPU, can also run on edge computing nodes (Babu *et al.*, 2022 ▸) or even in central computing facilities (Starostin *et al.*, 2022 ▸). For more integrated, routine solutions offered by the facilities themselves, edge computing is an attractive option in this context. Prioritizing the user’s requirements, it is advantageous for a facility to offer infrastructure that is exclusively available to the particular user group performing the experiment. Through collaborative efforts in the context of PaNOSC the VISA system based on OpenStack (see https://visa.readthedocs.io/) has been developed which essentially fulfills the needs described above.

Modern synchrotron beamlines are an ecosystem of distributed computing resources on their own. Therefore, a simple choice to establish communication between the beamline control software and the user-provided online data analysis resources is to rely on the beamline’s SCADA system (at ESRF: TANGO controls) for low-dimensional data (Sections SI-1 and SI-2 of the supporting information). Streaming options, however, become inevitable for high-rate, multidimensional data sources such as large area detector images.

**Figure 2:**
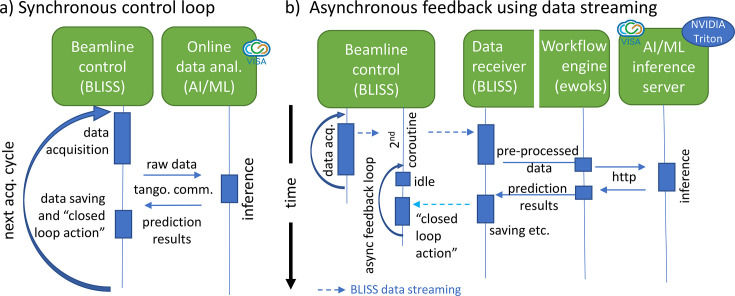
Illustration of timing and architecture of the implemented synchronous and asynchronous feedback loops. In (*a*), data acquisition, ML inference and feedback action follow each other strictly in time (synchronous over distributed system) while, in (*b*), acquisition and data analysis + feedback are separated in independent co-routines to decouple independent processes. Further direct communication with the ML model using TANGO controls is illustrated in (*a*) while in (*b*) an intermediate workflow system and an industrial inference server are used.

In Fig. 2 ▸ we illustrate the layout of two independently tested software configurations evaluated during the experiment presented. Figure 2 ▸(*a*) shows a fully *synchronous* acquisition and online analysis scheme relying on TANGO communication to transfer one-dimensional (1D) data [binned region-of-interest (ROI) intensities extracted from detector images, here done via LIMA (Petitdemange *et al.*, 2018 ▸)] to the online data analysis running on a user-controlled virtual machine provisioned via VISA where the ML inference takes place. After processing, ML results are made available in the main beamline control process and ‘closed loop action’ can be triggered. Once completed, another acquisition starts (see Section SI-2 of the supporting information).

Figure 2 ▸(*b*) shows a configuration that decouples the data acquisition and analysis + feedback into independent processes (asynchronous feedback) to maximize the data acquisition rates. Here, we use the streaming capabilities of *BLISS* to transfer reflectometry profiles into a workflow engine available at the beamline which in turn can communicate directly with an industrial AI inference server (here *Nvidia Triton* deployed on VISA hardware).

We emphasize again that for both described schemes no additional software needs to be installed into the beamline control software environment. The first, TANGO-based, approach largely benefits from the independence regarding the specific beamline control software solution and therefore its transferability to other facilities with a TANGO support layer on beamlines. Using industrial AI inference server in the second approach instead targets the use of standardized API interfaces commonly used in ML/AI.

### 2.3. ML methods

**Figure 3:**
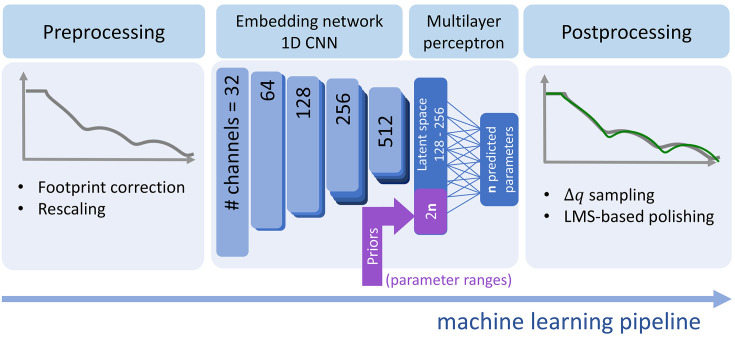
Sketch of the neural network architecture consisting of an embedding 1D CNN and prior injection into a combining multilayer perceptron. Here we use dynamic parameter ranges (min/max) for all free parameters. The *Q*-axis is to be defined prior to the training process, to be able to only provide 1D data (without *q* support vector). Postprocessing involves polishing of parameters predicted by ML by applying a traditional least-mean-squares fit.

Bringing ML into XRR data analysis is a community-wide effort also recognized by the Open Reflectometry Standards Organization (ORSO). There are several ML implementations with considerably different approaches, all being challenged by the ambiguity induced through the phase problem and the particularities of XRR signals, *e.g.* high dynamic range, sensitivity to marginal misalignment especially at the critical angle, and sensitivity to interpolation or insufficient sampling (Greco *et al.*, 2019 ▸; Munteanu *et al.*, 2023 ▸). This is addressed in existing implementations, *e.g.* by restricted parameter ranges (Doucet *et al.*, 2021 ▸; Mironov *et al.*, 2021 ▸; Greco *et al.*, 2021 ▸), a focus on identification of different families of SLD profiles based on symmetry (Carmona Loaiza & Raza, 2021 ▸), employing mixture density models (Kim & Lee, 2021 ▸), using variational autoencoders (Andrejevic *et al.*, 2022 ▸) or neural operators (Munteanu *et al.*, 2023 ▸) and convolutional neural networks (Aoki *et al.*, 2021 ▸). The approach chosen for this work distinguishes itself from previous implementations by allowing or accommodating prior knowledge (*e.g.* restricting parameter ranges) at inference time. This is achieved by implementing a 1D convolutional neural network (1D CNN) as an embedding network that is followed by a multiplayer perceptron (MLP) which combines prior information along with the XRR profile processed by the 1D CNN (Fig. 3 ▸). For further details regarding the specific network architecture used in the work we refer to previous works of the authors (Greco *et al.*, 2019 ▸, 2021 ▸, 2022 ▸; Mareček *et al.*, 2022 ▸; Munteanu *et al.*, 2023 ▸). For fast automated analysis of the measured XRR and Bragg reflection data, we rely on neural-network-based maximum-likelihood estimation (MLE). Compared with previous implementations, here we incorporate prior knowledge about the sample at a given time into the ML pipeline, thereby effectively mitigating uncertainty and constricting the range of potential solutions. For a comprehensive, detailed discussion of this approach, see Munteanu *et al.* (2023 ▸). Optimized implementation and the availability of dedicated GPU hardware during the experiment made the training process fast enough to allow for training from scratch, *e.g.* when adopting the sampling strategy during the beam time.

The reflectometry analysis aims at reconstructing the scattering length density (SLD) profile of the studied sample based on the measured reflectivity curve. Given an SLD profile, the corresponding theoretical curve can be swiftly calculated (Parratt, 1954 ▸). However, reversing this operation presents a challenge because of the inherent ambiguity that often allows for multiple, different SLD profiles to correspond to the same curve within the bounds of measurement uncertainty. Fundamentally, this is related to the famous phase problem of scattering (since only the intensity, not the phase, is recorded in the detector). Consequently, it is vital in reflectivity analysis to make use of the physical understanding of the investigated system in order to reduce the number of potential solutions and identify the correct one. In previous ML-based works with two-layered structures (Greco *et al.*, 2019 ▸; Hinderhofer *et al.*, 2023 ▸), we approached this task by effectively fixing most of the parameters characterizing the SLD profile and training the neural network to estimate only the three unfixed parameters – thickness, roughness and density of the top organic layer – anticipated to vary among the samples under study, with parameters of the silicon substrate with a silica top layer held constant. Expanding this method to accommodate a larger set of variable parameters, further techniques to address the ambiguity problem are needed (Munteanu *et al.*, 2023 ▸). In the present study, we build upon this approach and showcase two potential methods for integrating physical knowledge into the ML framework.

First, we propose to include the boundaries of the open parameters as an additional input to the neural network. As before, for each open parameter θ_*i*_ we designate broad ranges 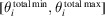 that determine the general scope of the neural network. Yet, in conjunction with these fixed ranges, we introduce *sample-dependent* ranges 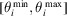 ( 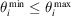 ; 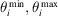 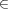 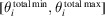 ) that impose constraints on the fitted parameters for each sample studied and are supplied as additional input to the neural network alongside the measured curve. This method effectively confines the solution space for a particular sample while maintaining extensive overall parameter ranges within a single neural network. In instances where a single solution exists within the provided sample-dependent boundaries, the inverse operation becomes well defined, enabling a precise fit. We note that this approach effectively combines the best of the two worlds: the flexibility of the parameter ranges of the conventional differential evolution method, and the speed of the neural network. Furthermore, for the real-time *in situ* analysis, it allows harvesting the information from the previous fits, as prior knowledge, so that the analysis of curves measured at different times for the same sample are no longer independent. For instance, during the growth experiment, we define the sample-dependent constraints on the growing film thickness based on the obtained result from a previous fit. This way, we leverage the physical understanding of the growth process (the thickness of the growing layer cannot decrease over time) and the experimental setup (thickness cannot increase too rapidly), without a specific growth model restriction as used by Mareček *et al.* (2022 ▸).

**Figure 4:**
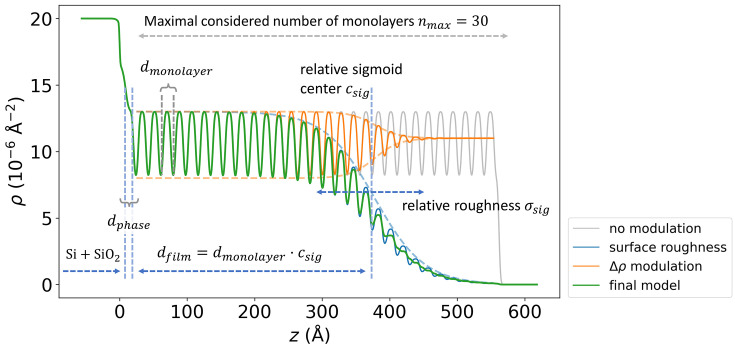
Parameterization of the periodic multilayer structure. The stacked layers share the same thickness *d*_monolayer_, roughnesses and SLD densities. Most of the parameters introduced are relative to *d*_monolayer_. Two sigmoid functions modulate the resulting periodic SLD profile. The position of the first sigmoid *c*_sig_ defines the number of monolayers and the corresponding thickness *d*_film_ = *d*_monolayer_ *c*_sig_, while its width σ_sig_ determines the surface roughness σ_film_ = σ_sig_ *d*_monolayer_. The second sigmoid modulates the contrast Δρ between the layers, resulting in smoother SLD profile at the surface.

The second method for integrating physical knowledge into the ML framework that we employ in this work is physics-based parameterization. In the case of ML-based reflectivity analysis, this approach was first introduced by Mareček *et al.* (2022 ▸), where the physics-based growth model allows the number of estimated parameters to be effectively reduced. In this work, we apply this approach to the case of periodic multilayer structures by implementing a physics-based parametrization of the SLD profile. The standard parameterization of the box model implies three parameters (or four parameters when including absorption) per single layer for each box (density, thickness, roughness). Given that a single molecular monolayer block is typically modeled by two layers, such parameterization would require up to 2 × 30 × 3 = 180 independently fitted parameters (when considering 30 monolayers) resulting in a 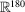 solution space. However, such parameters are largely correlated, because the monolayers consist of the same material, feature the same thickness, *etc*. To provide this information to the neural network, we introduce a set of 17 independently fitted or predicted parameters, that together define all the 180 parameters of the box model. Such parameterization is based on a physical understanding of monolayer structures and significantly decreases the task’s complexity (Mareček *et al.*, 2022 ▸). The parametrization is illustrated in Fig. 4 ▸ and is discussed in more detail by Munteanu *et al.* (2023 ▸).

We have developed a fast, GPU-accelerated module within the PyTorch framework that calculates reflectivity curves in order to train the neural networks utilized in the experiment in real time during the beam time. This module seamlessly integrates into the training process, enabling on-the-fly XRR simulations throughout the training phase. Consequently, we were able to quickly adjust the training settings (even during the beam time), eliminating the requirement for meticulous pre-planning. Note that we rely on preprocessing operations and the rich postprocessing pipeline introduced by Greco *et al.* (2021 ▸, 2022 ▸) that features *q* offset sampling and least-mean-squares (LMS) fit. A major addition to the postprocessing step of the previous works is the introduction of a new optional fitted parameter that models a linear change of thickness *during the measurement of a single curve* as we perform *in situ* and real-time experiments. Therefore, for a single scan, we take into account the time-dependent layer thickness *d*(*t*) and the time-dependent incidence angle corresponding to momentum transfer *q*(*t*). Together with other time-independent parameters, θ, *d*(*t*) and *q*(*t*) define the curve: *R* = *R*[*q*(*t*), *d*(*t*), θ]. A linear model of a growth process *d*(*t*) = *d*_0_ + *t*(Δ*d*/Δ*t*) is a very good approximation for the short duration of a quick real-time scan. Consequently, we fit two parameters *d*_0_ and Δ*d*/Δ*t* for each curve via LMS, using the neural network prediction as an initial guess for the parameter *d*_0_. The introduction of this time-dependent parameter is required to accurately fit XRR curves measured during the growth in the case of a fast growth process relative to the data acquisition time for a single XRR profile, as Kiessig fringes will be slightly narrower at high *q* as compared with low *q*.

### 2.4. Data handling

All data, including the extracted parameters of the online data analysis, have been stored in a fully correlated fashion, *i.e.* raw data alongside analysis results in NeXus HDF5, and are publicly available through the ESRF data portal (Pithan *et al.*, 2023 ▸).

Using the ESRF file-saving infrastructure (Bliss + NeXusWriter) enabled the visiting experimentalists to directly insert the online data analysis results into the data files published by the beamline (see supporting information for details). Furthermore, through integrating into the ESRF beamline software ecosystem, the online data analysis results could seamlessly be transferred into the accompanying electronic logbook, allowing a first overview of the experimental results ordered via timestamps.

### 2.5. Experiment

This study has been performed on the surface scattering branch of the ESRF ID10 beamline (Jankowski *et al.*, 2023 ▸). A beam energy of 17.0 keV and beam size of 30 µm × 30 µm were used. We use XRR, an established surface scattering technique, performed following standard procedures (Daillant & Gibaud, 2009 ▸; Tolan, 1999 ▸; Holý *et al.*, 1999 ▸; Seeck *et al.*, 2002 ▸). A user-supplied UHV sample environment has been installed in horizontal geometry. Molecular thin-film samples are prepared *in situ* using molecular beam deposition (Ritley *et al.*, 2001 ▸; Zykov *et al.*, 2017 ▸).

To achieve the objective of stabilizing and terminating the self-assembly, growth and crystallization characteristics of molecular thin-film studies *in situ*, a closed-loop control system has been implemented, leveraging the ML-based online analysis. This closed-loop control allows for autonomous experiments. In this particular study, the ML-based closed-loop system assumes control over the operation of two shutters, which involves covering either the substrate or the incoming molecular beam. To limit beam damage, which might occur for longer scan times at lower deposition rates, we reduced the X-ray flux to a level where there was no noticeable impact on Bragg peak intensities over the time of a growth run at our deposition rates.

In this study, molecular thin films of aluminium-tris(8-hydroxychinolin) (Alq3, C_27_H_18_AlN_3_O_3_), a frequently used component in organic light-emitting diodes, were grown to serve as an exemplary material system for amorphous molecular thin films (Mondal *et al.*, 2021 ▸). To demonstrate the online capabilities regarding the analysis of crystalline multilayer systems the organic semiconductor *N*,*N*′-dioctyl-3,4,9,10-perylendicarboximid (PTCDI-C_8_, C_40_H_42_N_2_O_4_) (Zykov *et al.*, 2017 ▸; Krauss *et al.*, 2008 ▸) was chosen for demonstration purposes. For details on the scientific background of these materials, we refer to Kowarik *et al.* (2008 ▸), Schreiber (2004 ▸) and Witte & Wöll (2004 ▸).

## 3. Results and discussion

To verify and test the technical solutions discussed above, we aim to grow molecular thin films of predefined thickness where the ML-based closed loop takes control over the termination of the growth process by closing the relevant deposition shutters. It became evident that it is crucial to provide prior knowledge about the film parameters (*e.g.* plausible film thickness ranges) as input of the ML-model to achieve robust fitting for a large number of consecutive scans. In this work priors were used to describe minimum and maximum boundaries for each parameter.

### 3.1. Discussion of ML in XRR and Bragg reflection fitting

**Figure 5:**
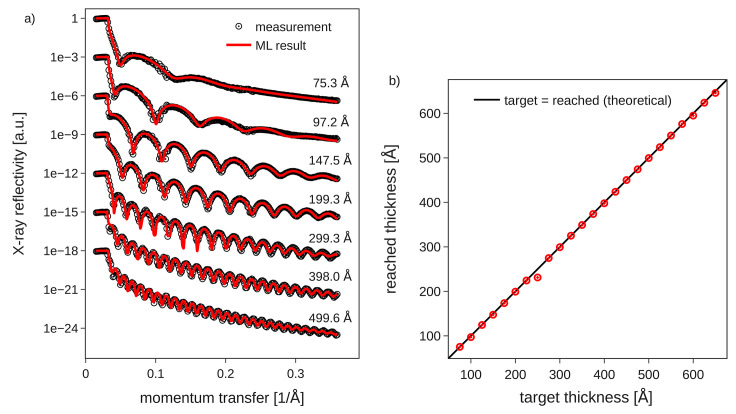
ML-controlled thin-film deposition. (*a*) Exemplary measurements and fits based on online data analysis. The experimental data are acquired using fast, real-time scans, thus the poor counting statistics around *q* ≃ 0.11 Å^−1^ are due to the limited number of absorber changes. (*b*) Target thicknesses in closed-loop operation versus actually measured thicknesses after the closed-loop feedback terminated the growth. Target thicknesses were defined in 25 Å steps starting from 75 Å. Profiles shown in (*a*) correspond to a subset of data points shown in (*b*).

To achieve good performance of ML predictions on XRR signals it is important to consider corrections such as *q*-shift (for slight misalignment) and subsequent fitting using an LMS algorithm as described by Greco *et al.* (2021 ▸). Furthermore, due to the *in situ* data-taking that is inherent to closed-loop feedback, the varying film thickness during a single scan must be taken into account if the scan speed is not fast compared with the deposition speed. We accommodate this additional effect as an additional parameter in the subsequent LMS fit as discussed above (snippet in Section SI-4 of the supporting information). In both examples of amorphous and multilayer thin films, prior information was taken into account for the online ML analysis result. In the multilayer fits physical knowledge was embedded in the parametrization of the electron density model and rather narrow training ranges of the ML model. In contrast, wide training ranges were used for the single-layer model, but prior information was taken into account by inputting it into the ML model to achieve a certain regularization of the results. Here, in the dataset presented in Fig. 5 ▸, through the use of priors, the expected film thickness was constrained to increase by at most 50 Å and decrease by no more than 25 Å for the ML-based real-time analysis. This still gives the model sufficient flexibility to predict changes during the film growth, but imposes weak boundaries based on the scan speed and deposition rate.

### 3.2. Single-layer fits (Kiessig oscillations)

In Fig. 5 ▸(*a*) we show exemplary reflectometry profiles acquired during thin-film growth of Alq3 using continuous scans (also known as *flyscans* or *fastscans*) together with the corresponding ML results of layer parameters. As can be seen from the presented footprint-corrected scans, we achieved a very good fit quality using the ML approach with priors. Both the period of Kiessig fringes as well as the roughness-induced damping of Kiessig fringes are correctly reproduced in the ML fits. Careful post-experiment data analysis did not yield any improvement of the fit quality with respect to online data analysis. The achieved high fit quality of the ML-based online data analysis is a prerequisite for achieving closed-loop control to terminate growth at the target thickness, but, due to the finite duration of the scans [45 s per XRR profile in Fig. 5 ▸(*a*)] and an average growth rate of 1 nm min^−1^, the target thickness may be reached during a scan. Therefore, we used a linear extrapolation of the thickness information of previous scans to automatically calculate the best moment to close the shutter, as yet another asynchronous process (see Section SI-3 of the supporting information). Figure 5 ▸(*b*) shows the result of the closed-loop deposition control for several target thicknesses between 80 Å and 640 Å. The target thicknesses are plotted on the *x*-axis, while the true film thicknesses at which the deposition was terminated are given on the *y*-axis. As expected for a functioning closed-loop control, the data indeed fall on the diagonal line, except for one outlier. Overall, the chosen target thicknesses could be reached within ±2 Å average accuracy.

### 3.3. Multilayer fits (Bragg reflections)

**Figure 6:**
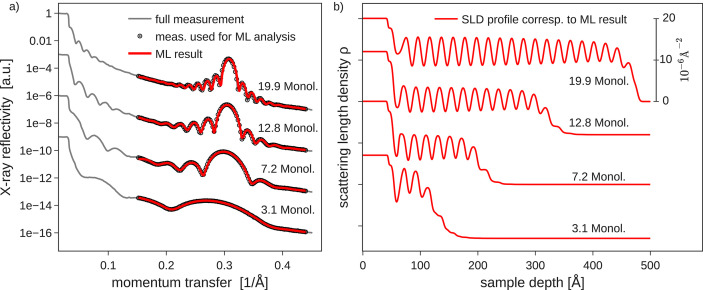
(*a*) ML-based fits of PTCDI-C_8_ multilayer structures (1 Monol. corresponds to ∼20 Å film thickness). As input for the ML model only points measured around the PTCDI-C_8_ Bragg peak were taken into account to enable faster data acquisition. (*b*) Scattering length density profiles corresponding to (*a*).

Not only amorphous thin-film structures but also multilayers of the molecule PTCDI-C_8_ were studied. By incorporating physical knowledge of the sample structure into the parametrization of the used ML model we are able to fit Laue fringes and the molecular Bragg peak that arises with increasing film thickness from molecular multilayers [Fig. 6 ▸(*a*)]. To speed up data acquisition, only a relatively short *q*-range centered around the molecular Bragg peak was repeatedly scanned while running in closed-loop mode. An initial ‘full’ XRR curve, including the total reflection edge, was used for signal normalization before activating the closed-loop operating mode.

**Figure 7:**
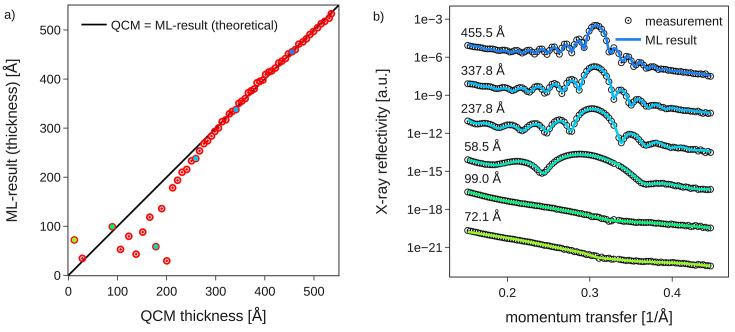
(*a*) Thin-film thickness extracted through the ML model in real time during the measurement compared with a quartz crystal microbalance (QCM) reference. For films with thickness above roughly 250 Å, *i.e.* 12 molecular monolayers, ML results are reliable. (*b*) Selected fits from the real-time dataset. Ambiguity in the thickness predictions for lower thicknesses mainly results from the absence of significant features in the investigated *q*-range.

Again, these scans around the Bragg reflection could be fitted by ML with high fidelity, reproducing the Bragg peak and the period of the Laue oscillations and their asymmetric intensity to the left and right of a Bragg reflection. The corresponding electron density profiles from the live fits are shown in Fig. 6 ▸(*b*), from which one can directly infer the number of deposited monolayers, as one oscillation of the scattering length density corresponds to one PTCDI-C_8_ monolayer. Comparing the film thickness from the ML fits of the Bragg region with the total thickness derived from a measurement of the deposition flux with a quartz crystal microbalance (QCM) one again finds reasonable agreement as shown in Fig. 7 ▸(*a*). For thicknesses above 250 Å the agreement is good. Some scatter is visible below this thickness, even though the quality ML fit of the individual reflectometry curves is good [Fig. 7 ▸(*b*)]. This particular example shows the difficulty of ambiguous XRR fits, where several sample structure models can fit a single X-ray curve. Also, note that the QCM measures total film thickness only if a constant sticking factor on the substrate and previously deposited molecular material are assumed. Further, the Laue oscillations do not correspond to the total film thickness but to the coherently ordered film thickness, so initial disorder in the crystal lattice may to some degree contribute to the observed deviations for low film thicknesses [additionally, of course, the potentially non-integer layer occupancy during growth may interplay as well (Rieutord *et al.*, 1989 ▸)]. Overall, we conclude that the ML results (live) of the coherent film thickness during data acquisition nicely match our detailed post-growth analysis and yield consistent data for larger film thickness of our sample system. This, in principle, enables closed-loop feedback, *e.g.* to target growth of a fixed number of crystalline lattice planes in a thin-film sample.

### 3.4. Robust feedback to control growth

Within the domain of ML-based closed-loop feedback, we emphasize the criticality of robust real-time analysis for successful operation. Especially in Fig. 5 ▸ the expected trend of increasing thickness from scan to scan during growth is clearly observed. The robustness of the ML results is especially important when facing the challenge on the control side that extracted parameters must be used to extrapolate the film thickness into the near future. Only robust fits can lead to a meaningful time-series, so that after extrapolation the autonomous growth termination can act at the moment the predefined film thickness is reached – which does not necessarily coincide with the end of a performed scan. In this study, the time resolution of the reflectivity measurements (repeat rate of scans) could be identified as the most critical bottleneck of the given setup. Therefore, the limited time resolution had to be addressed through an additional asynchronous process taking care of the temporal extrapolation and the triggering of actions in close-loop operation. Here *BLISS* is very well equipped for this kind of tasks through its tight integration of Gevent to enable co-routine operation (Bilenko & Madden, 2023 ▸). Through averaging several of the last ML-results (a.k.a. ‘predictions’), isolated slight outliers in the ML results could be tolerated and were not sufficient to invalidate the live feedback mechanism. Overall, the 2% thickness error proves the robust ML fitting, extrapolation and closed-loop feedback action.

### 3.5. Further integration potential

For the experiment conducted in the context of this work it was possible to store ML analysis results together with the original raw data and to interact with the facility-provided electronic logbook (ELN). However, ingesting machine-readable metadata into the facility data catalog remained difficult. In order to support and contribute to a thriving ecosystem of ML models for X-ray data analysis, *i.e.* physics-informed ML models and sample-system-specific ML models, it is of crucial importance that facilities provide – ideally standardized – interfaces and best practice guidelines on how externally developed code should interact with the respective beamline control and data storage systems. Initiatives similar to DAPHNE4NFDI (Barty *et al.*, 2023 ▸) are well suited for discussion in this context, since they bring together facilities and the user community, including experienced users, to jointly design research data infrastructure along a process chain all the way from the proposal and experiment to the fully analyzed and archived data.

## 4. Conclusions and outlook

In this study we established a complete closed-loop feedback cycle for controlling thin-film and crystal growth exclusively relying on real-time scattering data and online ML analysis. The presented scheme is well suited for a broader range of *in situ* and *in operando* experiments. Not only growth dynamics can be observed with X-rays but also processes in dynamic equilibrium where the information extracted from the scattering data itself can be used to stabilize the equilibrium. Specifically, for XRR this may for instance involve control over various liquid systems with a Langmuir trough as one possibility to prepare environments (*e.g.* lipid bilayers) with precise surface pressure to have stable conditions at the liquid–air interface. Here the ML approach for feedback directly relates to the film properties measured with XRR, whereas of course the sample environment (*e.g.* surface pressure of a Langmuir trough) may also be controlled externally. In a broader picture, we see a bright future to embed ML-based feedback loops also for other types of scattering experiments, *e.g.* involving electrochemical control over battery charging or control over electrochemical sample environment conditions or catalytic conditions in experiments on nanoparticles. It also extends to other synchrotron-based techniques (Chen *et al.*, 2021 ▸; Hinderhofer *et al.*, 2023 ▸), as well as neutron-based techniques, including in particular neutron reflectometry (Treece *et al.*, 2019 ▸).

With the focus on publicly available FAIR datasets hosted in facility data catalogs we see the potential of ML-based online data analysis in helping to make these datasets in catalogs ‘more fair and more reusable’. This is possible through enriching the currently archived raw dataset at least with the preliminary analysis results on-the-fly. Live ML X-ray data analysis has the ability to complete datasets with scientifically relevant machine-readable metadata as well as automated capture of scientific results in ELNs accompanying the dataset in the facility data catalog. We are convinced that the presented approach can contribute significantly to a more efficient use of beam time at large-scale facilities. We envision that integration of live data analysis and feedback loops with ML models will become more established at beamlines along the lines presented here. Then, facility users can use ML to observe live how experiments progress and also perform previously unattainable experiments with direct feedback and contribute to an ever-growing, meaningful X-ray dataset pool.

## 5. Data availability statement

The specific beamline integration Python scripts as well as the TANGO server embedding the ML model used in this study are available in the supporting information accompanying the published article. Data underlying this publication are available in the ESRF data repository (Pithan *et al.*, 2023 ▸). Experimental data used to prepare for the beam time are available – see Pithan *et al.* (2022 ▸). A reference ML model is available – see Greco (2022 ▸) and Munteanu *et al.* (2023 ▸).

## Supplementary Material

Supporting information for article. DOI: 10.1107/S160057752300749X/ju5054sup1.pdf

Raw data and results of online data analysis presented in this study: https://doi.org/10.15151/ESRF-DC-1249105707

## Figures and Tables

**Table 1:** Glossary of beamline control, open data and ML community initiatives and products

BLISS	High-level experiment control system developed at the ESRF providing a similar feature-set to *Bluesky*, predominately used at US facilities (https://gitlab.esrf.fr/bliss/bliss)
DAPHNE4NFDI	German, national big-data initiative embedded in NFDI (National Research Data Infrastructure) focusing on DAta from PHoton and Neutron Experiments
ExPaNDS	European Open Science Cloud (EOSC) Photon and Neutron Data Service; ExPaNDS is a federated structure of ten European national photon and neutron research institutions
HDF5	Container file format used for NeXus provided by the HDF Group
ICAT	Open source metadata management system designed for large facilities – ESRF is one facility, amongst others, using ICAT (https://icatproject.org)
LIMA	Library for Image Acquisition (ESRF) (https://lima1.readthedocs.io)
MLExchange	A web-based platform enabling exchangeable machine learning workflows for scientific studies
NeXus	A common data format, *e.g.* for neutron and X-ray science, defining community standards (https://www.nexusformat.org/)
ORSO	Open Reflectometry Standards Organization: an international, open effort to improve the scientific techniques of neutron and X-ray reflectometry (https://www.reflectometry.org)
PaNOSC	Photon and Neutron Open Science Cloud is the science cluster representing photon and neutron European research infrastructures providing and connecting related services to the European Open Science Cloud (EOSC)
SCADA	Supervisory Control And Data Acquisition system
SciCat	Community-developed metadata catalog, in use at several facilities including PSI, ESS, MAX IV, ALS as well as indiviual labs and research groups (https://scicatproject.github.io)
TANGO	SCADA system used at numerous scientific facilities including ESRF, ALBA, SOLEIL, DESY, ELI BEAMS and SAK-ZA. In the context of synchrotron facilities: device abstraction layer with comparable functionally to EPICS, another commonly used SCADA system in this kind of environments (https://www.tango-controls.org)

## Appendix A Glossary

See Table 1 ▸ for a glossary of beamline control, open data and ML community initiatives and products.
