# Supplementary material for: Closing the loop: autonomous experiments enabled by machine-learning-based online data analysis in synchrotron beamline environments
Source: J Synchrotron Radiat. 2023 Oct 17;30(Pt 6):1064–75. doi: 10.1107/S160057752300749X (PMC10624034; doi:10.1107/S160057752300749X)
Supplement: Supplementary file 1 [file s-30-01064-sup1.pdf]

Supporting Information - Closing the loop: Autonomous experiments enabled by machine-learning-based online data analysis in synchrotron beamline environments

Linus Pithan,[a*] Vladimir Starostin,[a] David Marecek,[b] Lukas Petersdorf,[c] Constantin Völter,[a] Valentin Munteanu,[a] Maciej Jankowski,[d] Oleg Konovalov,[d] Alexander Gerlach,[a] Alexander Hinderhofer,[a] Bridget Murphy,[c] Stefan Kowarik[b*] and Frank Schreiber [a*]

[a]*Universität Tübingen, Inst. für Angewandte Physik, Auf der Morgenstelle 10, 72076 Tübingen, Germany,* [b]*Universität Graz, Physikalische und Theoretische Chemie, Heinrichstraße 28, 8010 Graz, Austria ,* [c]*Universität Kiel, Inst. für Experimentelle und Angewandte Physik, Leibnizstraße 19, 24118 Kiel, Germany , and* [d]*ESRF, The European Synchrotron, 71 avenue des Martyrs, CS 40220, 38043 Grenoble Cedex 9, France . E-mail: linus.pithan@uni-tuebingen.de, stefan.kowarik@uni-graz.at, frank.schreiber]@uni-tuebingen.de*

machine learning; **reflectometry**; **xrr**; **nr**; **synchrotron**; **autonomous experiments**; **closed loop control**; **beamline control**

At the time of the experiment BLISS 1.10 and pytango 9.3 were available on the ID10 beamline.

SI-1. Tango device servers wrapping around ML Models

A tango device server with specific API to wrap around the ML models developed based on pytorch and tensorflow used in the context of this study.

```
from typing import Iterable
import numpy as np
from pprint import pprint

from tango.server import Device
from tango.server import run
from tango.server import attribute, command, device_property
from tango import AttrWriteType

# python code wrapping around pytorch model
# available on request or in follow-up publication
from reflectorch.inference import InferenceModel


def energy_to_wavelength(energy: float):
    """Conversion from photon energy (eV) to photon wavelength (angstroms)"""
    return 1.2398 / energy * 1e4


class TorchEvaluator(Device):
    preprocess_attributes = {
        "beam_width": np.float64,
        "sample_length": np.float64,
        "energy_keV": np.float64,
    }

    input_attributes = {
        "tth": [np.float64],
        "intensity": [np.float64],
        "transm": [np.float64],
        "priors": [[np.float64]],
        "count_time": [np.float64],
    }
    prediction_attributes = {
        "q": [np.float64],
        "q_interp": [np.float64],
        "refl": [np.float64],
        "refl_interp": [np.float64],
        "refl_predicted": [np.float64],
        "refl_predicted_polished": [np.float64],
        "parameters": [np.float64],
        "parameters_polished": [np.float64],
        "parameter_names": [str],
```

```
        "sld_profile": [np.float64],
        "sld_x_axis": [np.float64],
        "sld_profile_polished": [np.float64],
    }

    def __init__(self, *args, **kwargs):
        Device.__init__(self, *args, **kwargs)

        self._prefix_dict = dict()

        self._input_data = self._initialize_dynamic_attributes(
            "input", self.input_attributes
        )
        self._prediction_output = self._initialize_dynamic_attributes(
            "prediction", self.prediction_attributes
        )
        self._preprocess_data = self._initialize_dynamic_attributes(
            "preprocess", self.preprocess_attributes
        )
        self.inference_model = InferenceModel("l2q256_exp_3")

    @property
    def prediction_output(self):
        return self._prediction_output

    @command(dtype_out=int)
    def predict(self) -> dict:
        """
        blocking command that runs pytorch model inference
        """
        intensity = self._input_data["intensity"]
        scattering_angle = self._input_data["tth"]
        attenuation = self._input_data["transm"]
        priors = self._input_data["priors"]

        preprocess_params = {
            "wavelength": energy_to_wavelength(
                self._preprocess_data["energy_keV"] * 1000
            ),
            "beam_width": self._preprocess_data["beam_width"],
            "sample_length": self._preprocess_data["sample_length"],
        }

        self.inference_model.set_preprocessing_parameters(**preprocess_params)

        res_dict = self.inference_model.predict(
            intensity, scattering_angle, attenuation, priors,
        )

        pprint(res_dict)

        self.prediction_output["q"] = res_dict["q_values"]
        self.prediction_output["q_interp"] = res_dict["q_interp"]
        self.prediction_output["refl"] = res_dict["curve"]
        self.prediction_output["refl_interp"] = res_dict["curve_interp"
```

```
]
        self.prediction_output["refl_predicted"] = res_dict["curve_predicted"]
        self.prediction_output["parameters"] = res_dict["params"]
        self.prediction_output["sld_profile"] = res_dict["sld_profile"]
        self.prediction_output["sld_x_axis"] = res_dict["sld_x_axis"]
        self.prediction_output["parameter_names"] = res_dict["param_names"]

        self.prediction_output["refl_predicted_polished"] = res_dict["curve_polished"]
        self.prediction_output["parameters_polished"] = res_dict["params_polished"]
        self.prediction_output["sld_profile_polished"] = res_dict[
            "sld_profile_polished"
        ]

        return 0

    def _initialize_dynamic_attributes(self, prefix, attributes):
        """
        boiler plate code to achieve generic handling of data arrays and
        parameters that have to be passed into the tango server and back
        to the client
        """

        if prefix in self._prefix_dict:
            att_dict = self._prefix_dict[prefix]
        else:
            att_dict = dict()

        for k, v in attributes.items():
            att_type = None

            if isinstance(v, tuple):
                att_type = v[0]
                att_dict[k] = v[1]
            elif isinstance(v, Iterable):
                att_type = v
                att_dict[k] = []
            else:
                att_type = v
                att_dict[k] = None

            attr = attribute(
                name=prefix + "_" + k,
                dtype=att_type,
                access=AttrWriteType.READ_WRITE,
                fget=self._generic_read,
                fset=self._generic_write,
                max_dim_x=256,  # self.N_MAX_X,
                max_dim_y=256,  # self.N_MAX_Y,
                # fisallowed=self.generic_is_allowed,
            )
```

```
                self.add_attribute(attr)

            self._prefix_dict[prefix] = att_dict
            return self._prefix_dict[prefix]

        def _generic_read(self, attr):
            prefix, name = attr.get_name().split("_", 1)
            value = self._prefix_dict[prefix][name]
            # unlike a normal static attribute read, one has to modify the value
            # inside this attr object, rather than just returning the value
            attr.set_value(value)

        def _generic_write(self, attr):
            prefix, name = attr.get_name().split("_", 1)
            self._prefix_dict[prefix][name] = attr.get_write_value()


if __name__ == "__main__":
    run((TorchEvaluator,))
```

SI-2. Beamline / BLISS integration to run ML inference on remote tango device server and publish prediction results together with raw data

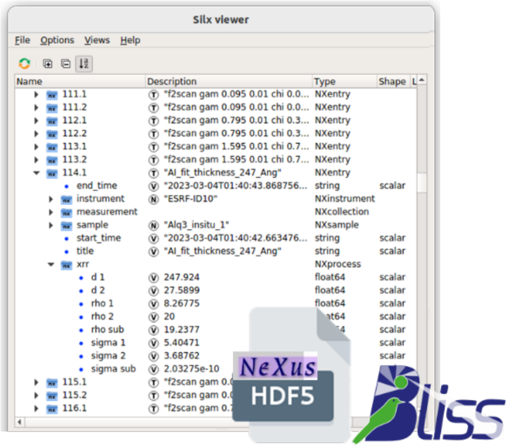

Fig. SI-1.: NeXus compliant data file generated by the beamline control system including results of the online data analysis.

Script that can be loaded as BLISS user macro into the beamline software environment without any additional external dependencies. The function predict_and_save takes a bliss scan object as input, runs ML inference and processes the results so that they are saved together with the raw data in a coherent fashion.

References:

https://bliss.gitlab-pages.esrf.fr/bliss/1.10.x/scan_group.html#sequences (accessed: 05.2023)

https://bliss.gitlab-pages.esrf.fr/bliss/1.10.x/data/data_scan_metadata.html (accessed: 05.2023)

```python
import numpy as np
from dataclasses import dataclass

from bliss.common import tango
from bliss import setup_globals
from bliss.scanning.group import Sequence
from bliss.scanning.chain import AcquisitionChannel
from bliss.scanning.scan_meta import USER_SCAN_META

# Add XRR Metadata entry to BLISS
if "xrr" not in USER_SCAN_META.categories_names():
    USER_SCAN_META.add_categories({"xrr"})
    USER_SCAN_META.xrr.set("xrr", {"@NX_class": "NXprocess"})


@dataclass
class Priors:
    d_top: tuple = (0.0, 1000.0)
    d_sio2: tuple = (10.0, 30.0)

    sigma_top: tuple = (0.0, 50.0)
    sigma_sio2: tuple = (
        0.0,
        10.0,
    )
    sigma_si: tuple = (
        0.0,
        2.0,
    )

    rho_top: tuple = (7.0, 14.0)
    rho_sio2: tuple = (19, 23)
    rho_si: tuple = (19, 21)
    max_d_change: float = 5.0
    fit_growth: bool = True

    def to_arr(self):
        return np.array(
            [
                self.d_top,
                self.d_sio2,
                self.sigma_top,
                self.sigma_sio2,
                self.sigma_si,
```

```
                self.rho_top,
                self.rho_sio2,
                self.rho_si,
            ]
        )


@dataclass
class PreprocessParams:
    energy_keV: float = 17.0
    beam_width: float = 0.03
    sample_length: float = 9.0


def predict_and_save(
    scans, priors=None, preprocess: PreprocessParams = None,
):
    """
    takes a list of scans that will be concatenated to a single XRR profile,
    preforms basic background correction, pushes data via TANGO into the ML model,
    triggers inference in the TANGO device, retrieves prediction results and
    publishes results via BLISS to be saved together with original scans in
    the NeXus hdf5 file produced by the BLISS NeXusWriter
    """

    # specify the tango uri of the device server embedding the ML-Model
    tango_uri_or_proxy = ("//VISA-HOST.esrf.fr:PORT/PATH/TO/TANGO-ML-DEVICE",)

    # specify counters that should be used to collect the data from the scan object
    TTH = setup_globals.gam  # gam: angle of detector axis
    CROI = "pilatus300k:roi_counters:roi2_sum"  # ROI with specular signal
    BGROI1 = (
        "pilatus300k:roi_counters:roi3_sum"  # ROI left of CROI (half the size of CROI)
    )
    BGROI2 = (
        "pilatus300k:roi_counters:roi4_sum"  # ROI right of CROI (half the size of CROI)
    )
    TRANSM = "autof_eh1:transm"  # transmission of Autoabsorber

    # extract detector angle from scans
    tth_data = np.concatenate(np.array([x.get_data(TTH) for x in scans]))

    # extract transmission of attenuators from scans
    transm_data = np.concatenate(np.array([x.get_data(TRANSM) for x in scans]))

```

```python
    # extract measured intensities from scans and perform basic background correction
    intens_data = np.concatenate(
        np.array(
            [x.get_data(CROI) - x.get_data(BGROI1) - x.get_data(BGROI2) for x in scans]
        )
    )

    # establish communication with tango device that is linked to ML model
    ml_device = tango.DeviceProxy(tango_uri_or_proxy)

    # deal with priors and preprocessing parameters
    priors = priors or Priors()
    preprocess = preprocess or PreprocessParams()

    # push data to tango device
    ml_device.input_tth = tth_data
    ml_device.input_transm = transm_data
    ml_device.input_intensity = np.clip(intens_data, 0, np.infty)
    ml_device.input_count_time = [0]
    ml_device.input_priors = priors.to_arr()
    ml_device.postprocess_max_d_change = priors.max_d_change
    ml_device.postprocess_fit_growth = priors.fit_growth
    ml_device.preprocess_beam_width = preprocess.beam_width
    ml_device.preprocess_sample_length = preprocess.sample_length
    ml_device.preprocess_energy_keV = preprocess.energy_keV

    # run inference (here built to run synchronous in blocking fashion)
    ml_device.set_timeout_millis(5000)
    ml_device.predict()

    # retrieve predicted parameters
    # polished parameters refers to results of subsequent LMS fit after inference
    polished_params = ml_device.prediction_parameters_polished
    param_names = ml_device.prediction_parameter_names

    # create summary metadata structure that is to be published via BLISS
    info = {"xrr": dict(zip(param_names, polished_params))}

    # creates a new entry in the hdf5 file generated by BLISS and redis DB for online access
    seq = Sequence(
        title=f"AI_fit_thickness_{int(polished_params[0])}_Ang", scan_info=info
    )

    # specify additional data entries that should appear under "measurement" in the h5 file
    seq.add_custom_channel(AcquisitionChannel("raw_tth", float, ()))
    seq.add_custom_channel(AcquisitionChannel("corrected_intensity", float, ()))
    seq.add_custom_channel(AcquisitionChannel("raw_q", float, ()))
```

```python
    seq.add_custom_channel(AcquisitionChannel("predicted_intensity", float, ()))
    seq.add_custom_channel(AcquisitionChannel("predicted_q", float, ()))
    seq.add_custom_channel(AcquisitionChannel("params", float, ()))
    seq.add_custom_channel(AcquisitionChannel("params_names", str, ()))
    seq.add_custom_channel(AcquisitionChannel("polished_intensity", float, ()))
    seq.add_custom_channel(AcquisitionChannel("polished_params", float, ()))

    # add references to the raw data scans as well as process data and analysis results
    with seq.sequence_context() as scan_seq:
        for scan in scans:
            scan_seq.add(scan)

        seq.custom_channels["raw_tth"].emit(tth_data)
        seq.custom_channels["corrected_intensity"].emit(ml_device.prediction_refl)
        seq.custom_channels["raw_q"].emit(ml_device.prediction_q)
        seq.custom_channels["predicted_intensity"].emit(
            ml_device.prediction_refl_predicted
        )
        seq.custom_channels["params"].emit(ml_device.prediction_parameters)
        seq.custom_channels["params_names"].emit(ml_device.prediction_parameter_names)
        seq.custom_channels["polished_intensity"].emit(
            ml_device.prediction_refl_predicted_polished
        )
        seq.custom_channels["polished_params"].emit(polished_params)

    # return polished_params e.g. for closed loop feedback
    return polished_params
```

SI-3. Asynchronous BLISS integration to receive events via the BLISS data streaming API

The code snippet below represents basically a consumer of events emitted via the event based data streaming api of BLISS and publishes produced results using a beacon channel.

References: `https://bliss.gitlab-pages.esrf.fr/bliss/1.10.x/data/data_redis_api_low.html` (accessed: 05.2023)

`https://bliss.gitlab-pages.esrf.fr/bliss/1.10.x/beacon_channels.html` (accessed: 05.2023)

```python
from bliss.data.node import get_session_node
from bliss.config.channels import Channel

# create BLISS Channel with unique name in the scope of the beamline
# to publish predictions results via beacon / redis
last_film_thickness = Channel("last_film_thickness", default_value=None)


def listen_scans_of_session(session):
    """
    script that can run in any bliss process
    (independent oft the process producing the data & controlling the hardware)
    takes as input argument the name of the bliss session publishing the data
    to the beamline beacon server / redis
    """

    session_node = get_session_node(session)
    print(f"Listening to {session}")

    received_scans = []
    for event_type, node, event_data in session_node.walk_on_new_events(
        include_filter=["scan"]
    ):
        # this loop deals with the event based data streaming api of BLISS
        #
        if event_type == event_type.NEW_NODE:
            print(f"new scan", node.db_name)

        elif event_type == event_type.END_SCAN:
            print(node.db_name)

            params = node.info.get("fastfit")
            if params.get("xrr_scan_group_first", False):
                received_scans = [node]
                print("add scan")
            if params.get("xrr_scan_group_last", False):
                received_scans.append(node)
                print("add scan and process")
                process_scans(received_scans)


def process_scans(scans):
    """
    Run ML inference
    """

    # missing here: code similar to synchronous version in example above
    # interacting with tango or triton server to retrieve prediction

    # ...
```

IUCr macros version 2.1.17: 2023/05/02

```
    thickness = prediction["parameters_polished"][0]

    # publish e.g. film thickness via bliss channel & beacon
    # to make it available to all bliss processes running on the
   beamline
    last_film_thickness.value = thickness

    print("######### predicted thickness", thickness)
```

SI-4. Postprocessing gradient-descent fit with optional growth rate parameter.

The functions below are used to perform a postprocessing polishing operation by fitting the measured curve via gradient descent initialized based on AI result. Optionally, it allows fitting the linear change of the thickness of the top layer as a function of q for curves measured *in situ* during growth. Further details may be found in (Munteanu *et al.*, 2023).

References:

Munteanu, V., Starostin, V., Greco, A., Pithan, L., Gerlach, A., Hinderhofer, A., Kowarik, S. Schreiber, F. (2023). Neural network analysis of neutron and X-ray reflectivity data: incorporating prior knowledge for tackling the phase problem.

```
import numpy as np
from scipy.optimize import minimize, curve_fit

def abeles_np(
        q: np.ndarray,
        thickness: np.ndarray,
        roughness: np.ndarray,
        sld: np.ndarray):

    c_dtype = np.complex128 if q.dtype is np.float64 else np.complex64

    if q.ndim == thickness.ndim == roughness.ndim == sld.ndim == 1:
        zero_batch = True
    else:
        zero_batch = False

```

```python
    thickness = np.atleast_2d(thickness)
    roughness = np.atleast_2d(roughness)
    sld = np.atleast_2d(sld)
    batch_size, num_layers = thickness.shape
    sld = np.concatenate([np.zeros((batch_size, 1)).astype(sld.dtype), sld], -1)[:, None]
    thickness = np.concatenate([np.zeros((batch_size, 1)).astype(thickness.dtype), thickness], -1)[:, None]
    roughness = roughness[:, None] ** 2

    sld = sld * 1e-6 + 1e-30j

    k_z0 = (q / 2).astype(c_dtype)
    if len(k_z0.shape) == 1:
        k_z0 = k_z0[None]
    if len(k_z0.shape) == 2:
        k_z0 = k_z0[..., None]
    k_n = np.sqrt(k_z0 ** 2 - 4 * np.pi * sld)
    k_n, k_np1 = k_n[..., :-1], k_n[..., 1:]

    beta = 1j * thickness * k_n

    exp_beta = np.exp(beta)
    exp_m_beta = np.exp(-beta)
    rn = (k_n - k_np1) / (k_n + k_np1) * np.exp(- 2 * k_n * k_np1 * roughness)

    c_matrices = np.stack([
        np.stack([exp_beta, rn * exp_m_beta], -1),
        np.stack([rn * exp_beta, exp_m_beta], -1),
    ], -1)
    c_matrices = np.moveaxis(c_matrices, -3, 0)
    m, c_matrices = c_matrices[0], c_matrices[1:]
    for c in c_matrices:
        m = m @ c

    r = np.abs(m[..., 1, 0] / m[..., 0, 0]) ** 2
    r = np.clip(r, None, 1.)

    if zero_batch:
        r = r[0]
    return r


def standard_restore_params(fitted_params) -> dict:
    num_layers = (fitted_params.size - 2) // 3
    return dict(
        thickness=fitted_params[:num_layers],
        roughness=fitted_params[num_layers:2 * num_layers + 1],
        sld=fitted_params[2 * num_layers + 1:],
    )


def mse_loss(curve1, curve2):
    return np.sum((curve1 - curve2) ** 2)
```

```python

def standard_refl_fit(
        q: np.ndarray, curve: np.ndarray,
        init_params: np.ndarray,
        bounds: np.ndarray = None,
        refl_generator=abeles_np,
        restore_params_func=standard_restore_params,
        scale_curve_func=np.log10,
        **kwargs):

    if bounds is not None:
        kwargs['bounds'] = bounds
        init_params = np.clip(init_params, *bounds)

    res = curve_fit(
        get_scaled_curve_func(
            refl_generator=refl_generator,
            restore_params_func=restore_params_func,
            scale_curve_func=scale_curve_func,
        ),
        q, scale_curve_func(curve),
        p0=init_params, **kwargs
    )

    curve = refl_generator(q, **restore_params_func(res[0]))
    return res[0], curve


def get_fit_with_growth(
        q: np.ndarray, curve: np.ndarray,
        init_params: np.ndarray,
        bounds: np.ndarray = None,
        init_d_change: float = 0.,
        max_d_change: float = 30.,
        scale_curve_func=np.log10,
        **kwargs):

    init_params = np.array(list(init_params) + [init_d_change])
    if bounds is not None:
        bounds = np.concatenate([bounds, np.array([0, max_d_change])
    [..., None]], -1)

    params, curve = standard_refl_fit(
        q, curve, init_params, bounds, refl_generator=
    refl_with_changing_params,
        restore_params_func=get_restore_params_with_growth_func(q_size=
    q.size, d_idx=0),
        scale_curve_func=scale_curve_func, **kwargs
    )
    params[0] += params[-1] / 2
    return params, curve


def fit_refl_curve(q: np.ndarray, curve: np.ndarray,
                   init_params: np.ndarray,
                   bounds: np.ndarray = None,
```

```
                    refl_generator=abeles_np,
                    restore_params_func=standard_restore_params,
                    scale_curve_func=np.log10,
                    **kwargs
                    ) -> np.ndarray

    fitting_func = get_fitting_func(
        q=q, curve=curve,
        refl_generator=refl_generator,
        restore_params_func=restore_params_func,
        scale_curve_func=scale_curve_func,
    )

    res = minimize(fitting_func, init_params, bounds=bounds, **kwargs)

    if not res.success:
        import warnings
        warnings.warn(f"Minimization did not converge.")
    return res.x


def get_scaled_curve_func(
        refl_generator=abeles_np,
        restore_params_func=standard_restore_params,
        scale_curve_func=np.log10):

    def scaled_curve_func(q, *fitted_params):
        fitted_params = restore_params_func(np.asarray(fitted_params))
        fitted_curve = refl_generator(q, **fitted_params)
        scaled_curve = scale_curve_func(fitted_curve)
        return scaled_curve

    return scaled_curve_func


def get_fitting_func(
        q: np.ndarray,
        curve: np.ndarray,
        refl_generator=abeles_np,
        restore_params_func=standard_restore_params,
        scale_curve_func=np.log10,
        loss_func=mse_loss):

    scaled_curve = scale_curve_func(curve)

    def fitting_func(fitted_params):
        fitted_params = restore_params_func(fitted_params)
        fitted_curve = refl_generator(q, **fitted_params)
        loss = loss_func(scale_curve_func(fitted_curve), scaled_curve)
        return loss

    return fitting_func


def restore_masked_params(fixed_params, fixed_mask):
    def restore_params(fitted_params) -> dict:
```

```python
        params = np.empty_like(fixed_mask).astype(fitted_params.dtype)
        params[fixed_mask] = fixed_params
        params[~fixed_mask] = fitted_params
        return standard_restore_params(params)

    return restore_params


def base_params2growth(base_params: dict, d_shift: np.ndarray, d_idx: int = 0) -> dict:
    d_init = base_params['thickness'][None]
    q_size = d_shift.size
    d = d_init.repeat(q_size, 0)
    d[:, d_idx] = d[:, d_idx] + d_shift

    roughness = np.broadcast_to(base_params['roughness'][None], (q_size, base_params['roughness'].size))
    sld = np.broadcast_to(base_params['sld'][None], (q_size, base_params['sld'].size))

    return {
        'thickness': d,
        'roughness': roughness,
        'sld': sld,
    }


def get_restore_params_with_growth_func(q_size: int, d_idx: int = 0):
    def restore_params_with_growth(fitted_params) -> dict:
        fitted_params, delta_d = fitted_params[:-1], fitted_params[-1]
        base_params = standard_restore_params(fitted_params)
        d_shift = np.linspace(0, delta_d, q_size)
        return base_params2growth(base_params, d_shift, d_idx)

    return restore_params_with_growth


def refl_with_changing_params(q: np.ndarray, **kwargs):
    return abeles_np(q[..., None], **kwargs).flatten()
```
